# Converging Pathways between Metabolic Dysfunction–Associated Steatotic Liver Disease (MASLD) and Diabetes in Children

**DOI:** 10.3390/ijms25189924

**Published:** 2024-09-14

**Authors:** Maria Felicia Faienza, Ilaria Farella, Mohamad Khalil, Piero Portincasa

**Affiliations:** 1 Pediatric Unit, Department of Precision and Regenerative Medicine and Ionian Area (DiMePre-J), Medical School, University of Bari “Aldo Moro”, 70124 Bari, Italy; 2 Clinica Medica “A. Murri”, Department of Precision and Regenerative Medicine and Ionian Area (DiMePre-J), Medical School, University of Bari “Aldo Moro”, 70124 Bari, Italy; ilafarella@yahoo.com (I.F.); mohamad.khalil@uniba.it (M.K.)

**Keywords:** liver, steatosis, obesity, non-alcoholic fatty liver disease, metabolic-associated (dysfunction) steatotic liver disease, magnetic resonance imaging

## Abstract

In the past thirty years, childhood obesity rates have risen significantly worldwide, affecting over 340 million children in affluent nations. This surge is intricately tied to metabolic disorders, notably insulin resistance, type 2 diabetes mellitus (T2DM), and the continually evolving spectrum of metabolic-associated (dysfunction) steatotic liver disease (MASLD). This review underscores the alarming escalation of childhood obesity and delves comprehensively into the evolving and dynamic changes of nomenclature surrounding diverse conditions of hepatic steatosis, from the initial recognition of non-alcoholic fatty liver disease (NAFLD) to the progressive evolution into MASLD. Moreover, it emphasizes the crucial role of pediatric endocrinologists in thoroughly and accurately investigating MASLD onset in children with T2DM, where each condition influences and exacerbates the progression of the other. This review critically highlights the inadequacies of current screening strategies and diagnosis, stressing the need for a paradigm shift. A proposed solution involves the integration of hepatic magnetic resonance imaging assessment into the diagnostic arsenal for children showing insufficient glycemic control and weight loss post-T2DM diagnosis, thereby complementing conventional liver enzyme testing. This holistic approach aims to significantly enhance diagnostic precision, fostering improved outcomes in this vulnerable high-risk pediatric population.

## 1. Introduction

Over the last three decades, there has been a twofold to threefold increase in childhood obesity rates in high-income countries. According to the 2017 data from the WHO Global Health Observatory, obesity impacts over 340 million children and adolescents aged 5–19 years [1]. The rise in obesity prevalence parallels the increase in other metabolic disorders such as expanded visceral adiposity, type 2 diabetes (T2DM), dyslipidemia, liver steatosis, hypertension, atherosclerosis [2,3], and cholesterol cholelithiasis [4]. Obesity is associated with ongoing sub-clinical chronic low-grade inflammation in various tissues, including adipose tissue, skeletal muscle, liver, pancreatic islets, intestine, and brain [5]. The inflammation involves both innate and adaptive immune cells and acts as a causal link between obesity and its metabolic-dysfunction-associated complications such as insulin resistance and T2DM. The inflammatory background in obesity was first suspected after a report of elevated TNFα levels in adipose tissue [6]. The precise molecular mechanisms through which inflammation in adipose tissue contributes to insulin resistance are not thoroughly understood and may involve multiple factors. Various immune cells, particularly macrophages and T cells [7,8,9], likely contribute to insulin resistance associated with obesity. These cells release type 1 cytokines such as TNFα, interleukin (IL)-1β, and IFNγ which can negatively regulate metabolism and induce insulin resistance in various cell types, including adipocytes and skeletal muscle myocytes, through paracrine or endocrine effects. The cytokines exert their effects by interacting with receptors in the cells, triggering a cascade of intracellular signaling pathways. These pathways, in turn, compromise insulin signaling and lead to the development of insulin resistance in the affected cells [10]. Insulin resistance and chronic inflammation play pivotal roles in the development of T2DM, contributing to liver steatosis, and vice versa, via the accumulation of free fatty acids as triglyceride droplets in the liver cells, thus causing lipotoxicity, inflammation, and fibrosis [11].

In this context, liver steatosis can develop as the organ manifestation of metabolic dysfunctions. The first definition of non-alcoholic fatty liver disease (NAFLD) refers to the presence of steatosis by imaging or liver biopsy in individuals with no alcohol consumption or a daily alcohol consumption of ≤20 g in females and ≤30 g in males and no other cause of chronic liver disease [12]. NAFLD is a prevalent chronic liver disease, affecting more than 30% of the global population worldwide [13,14] and has become the predominant cause of chronic liver disease in adults [15,16]. NAFLD is usually a silent condition, but, in a subgroup of patients, NAFLD can progress towards non-alcoholic steatohepatitis (NASH), advanced fibrosis, liver cirrhosis, and, ultimately, to hepatocellular carcinoma [16]. The term NAFLD, however, does not accurately reflect the current understanding of disease drivers, while there is a growing awareness about the close link between NAFLD and metabolic dysfunctions/metabolic syndrome [17,18]. This situation has recently promoted the shift of terminology from NAFLD to metabolic dysfunction–associated fatty liver disease (MAFLD) in 2020 [17,18] and metabolic dysfunction–associated steatotic liver disease (MASLD) in 2023 within the overarching term of steatotic liver disease (SLD), encompassing several types of liver steatosis [14,19].

This review will discuss the pediatric endocrinologist’s point of view when screening pediatric patients with T2DM, to detect the potential onset of MASLD more thoroughly and accurately. We will continue using the acronyms NAFLD/NASH when discussing studies designed before the change of nomenclature to MASLD. Otherwise, MASLD/MASH will become the terms of reference.

## 2. Diagnostic Criteria of Liver Steatosis along with Shift of Terminology

**Figure 1:**
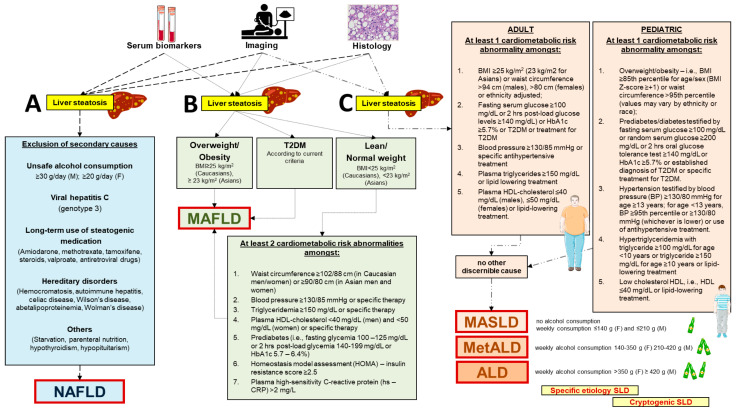
Diagnostic criteria related to novel nomenclatures indicative of diverse clinical entities associated with initial steatotic liver disease. In the presence of liver steatosis testified by specific serum biomarkers, imaging (usually liver ultrasound diagnosing “bright” hyperechoic liver), or liver biopsy and histology. (A) The diagnosis of non-alcoholic fatty liver disease (NAFLD) is based on the exclusion of steatogenic causes [12]. (B) The diagnosis of metabolic dysfunction–associated fatty liver disease (MAFLD) is based on the presence of overweight/obesity or type 2 diabetes or, in lean/normal weight individuals, accumulating at least two out of seven cardiometabolic risk abnormalities [17,18]. (C) The diagnosis of metabolic dysfunction–associated steatotic liver disease (MASLD) is part of the initial condition of steatotic liver disease (SLD). The pathway of MASLD takes into account the adult and pediatric age and requires at least one out of five cardiometabolic risk abnormalities and no other discernible cause. The criterion of weekly alcohol consumption becomes important to further classify the steatotic liver as MASLD (no or little alcohol consumption), combined metabolic dysfunction–associated alcoholic liver disease (MetALD), or pure alcoholic liver disease (ALD). The specific etiologies of steatotic liver or cryptogenic liver disease need also to be considered according to specific cases when finalizing the diagnosis of MASLD [19,20,25]. Partly created with BioRender.com (accessed on 1 August 2024).

The change in terminology, i.e., NAFLD to MAFLD to MASLD, will likely improve the classification of patients, disease awareness, the terminology comprehension concerning the involved pathophysiological aspects (not only a diagnosis of exclusion such as for NAFLD), and, hopefully, pave the way for more personalized therapeutic approaches [20]. The stigmatization of terms such as “non-alcoholic” and “fatty” will be overcome as well [17,18,19]. Following the consensus document, comparative studies by registries, population studies, community cohorts, and primary care settings point to an almost complete overlap of populations when using the terminology MASLD with respect to NAFLD [21,22,23], with MASLD requiring the presence of at least one of five cardiometabolic risk factors in the context of hepatic steatosis [14]. MASLD does not impact the characterization of fibrotic severity or the definition of steatohepatitis (based on the presence of liver inflammation and hepatocellular injury, i.e., ballooning of hepatocytes, with or without fibrosis), and, therefore, MASH can replace NASH without altering the predicted sequence to cirrhosis [24] (Figure 1).

Diagnostic criteria related to the novel nomenclatures differ in the case of NAFLD, MAFLD, or MASLD (Figure 1). For both NAFLD and MAFLD, the initial diagnosis of “liver steatosis” is achieved by blood biomarkers, imaging, or histology after liver biopsy. For MASLD, the starting point is the initial diagnosis of “steatotic liver”, which requires either imaging or histology. The following diagnostic pathways also differ with the three nomenclatures. The presence of NAFLD is based on exclusion of major steatogenic causes [12]. The diagnosis of MAFLD is more focused on metabolic aspects and is based on the presence of overweight/obesity or type 2 diabetes. In addition, in the case of lean/normal weight individuals, MAFLD is diagnosed in those accumulating at least two out of seven cardiometabolic risk abnormalities [17,18]. The diagnosis of MASLD takes into account the adult and pediatric age and requires at least one out of five cardiometabolic risk abnormalities. Other discernible causes must be absent. The criterion of weekly alcohol consumption becomes important to further classify the steatotic liver as MASLD (no or little alcohol consumption), metabolic dysfunction–associated alcoholic liver disease (MetALD), or pure alcoholic liver disease (ALD). The specific etiologies of steatotic liver or cryptogenic liver disease need also to be considered according to specific cases when finalizing the diagnosis of MASLD. In the absence of overt cardiometabolic criteria, the exploration of alternative causes is essential to reach the diagnosis of a specific etiology. If none is identified, the steatotic liver disease is termed cryptogenic. If additional factors contributing to steatosis are identified, a combined etiology is recognized [19,20,25]. The diagnostic criteria for metabolic-associated steatotic liver disease (MASLD) often involve the use of serological markers, imaging techniques, and liver biopsy. In addition to these diagnostic criteria, it is important to mention the use of non-invasive scoring systems such as the FIB-4, NAFLD Fibrosis Score (NFS), and the Aspartate Aminotransferase to Platelet Ratio Index (APRI). These tools are regularly used in clinical practice to estimate the presence and severity of liver fibrosis in patients with hepatic steatosis. For example, FIB-4 is a scoring system that combines age, AST, ALT values, and platelet count, proving useful in distinguishing patients with advanced fibrosis from those without significant fibrosis, thereby reducing the need for invasive liver biopsies [26].

### 2.1. Refining the Diagnosis of MASLD

#### 2.1.1. Adults

According to the current nomenclature following the Delphi consensus [19], the overarching SLD diagnosed by imaging or histology encompasses subcategories that include the following:

MASLD (i.e., liver steatosis, at least one cardiometabolic risk factor, no other discernible cause, no alcohol consumption or a weekly consumption ≤ 140 g in females and ≤210 g in males). In adult populations, the initial diagnosis of SLD requires the exact analysis of cardiometabolic criteria to reach the final diagnosis of MASLD or MetALD. The abnormalities are the following:

1. Body-mass index (BMI) ≥ 25 kg/m^2^ (23 kg/m^2^ for Asians) or waist circumference > 94 cm (males), >80 cm (females) or ethnicity-adjusted;
2. Fasting serum glucose ≥ 100 mg/dL or 2-hrs post-load glucose levels ≥ 140 mg/dL or HbA1c ≥ 5.7% or T2DM or treatment for T2DM;
3. Blood pressure ≥ 130/85 mmHg or specific antihypertensive treatment;
4. Plasma triglycerides ≥ 150 mg/dL or lipid-lowering treatment;
5. Plasma HDL-cholesterol ≤ 40 mg/dL (males), ≤50 mg/dL (females), or lipid-lowering treatment.

MetALD, i.e., an overlap of MASLD and ALD, i.e., liver steatosis, no other discernible cause, and intermediate weekly alcohol consumption of 140–350 g in females and 210–420 g in males, with a continuum ranging from MASLD-predominant to ALD-predominant types.

ALD, i.e., liver steatosis with weekly alcohol consumption > 350 g in females and ≥420 g in males).

Specific-etiology SLD (i.e., drug-induced liver injury (DILI), monogenic diseases such as lysosomal acid lipase deficiency (LALD), Wilson disease, hypobetalipoproteinemia, inborn error of metabolism, and miscellaneous, such as Hepatitis C virus, malnutrition, celiac disease).

Cryptogenic SLD, a label prone to re-classification in the future, as long as further diagnostic entities are reached. Importantly, the diagnosis of SLD must be re-assessed periodically to rule out incoming findings suggesting novel diagnoses.

For MASLD, the concept of an affirmative diagnosis allows for the coexistence of mixed forms of liver disease, e.g., MASLD plus viral hepatitis or autoimmune hepatitis.

#### 2.1.2. Children and Adolescents

MAFLD has also become the most common cause of liver disease in children. This phenotype will likely benefit from early interventions aimed at the treatment of liver steatosis, obesity, and metabolic abnormalities, therefore reducing the long-term consequences of metabolic, cardiovascular, and liver complications. Aspects related to a child’s and family’s quality of life need also to be considered [27]. In this respect, lifestyle changes consisting of dietary interventions, physical activity, and nutritional and psychological counseling have been shown to improve body-mass index (BMI), aminotransferase levels, and hepatic steatosis in children with MAFLD [28]. Diagnostic criteria for the pediatric MASLD area are also detailed in the Delphi consensus paper and are very close to the adult ones [19]. Following the first diagnosis of steatotic liver on imaging or liver biopsy, at least one specific cardiometabolic risk factor out of five must be present, i.e.,:

1. Overweight/obesity, i.e., BMI ≥ 85th percentile for age/sex (BMI Z-score ≥ +1) or waist circumference > 95th percentile (values may vary by ethnicity or race).
2. Prediabetes/diabetes testified by fasting serum glucose ≥ 100 mg/dL or random serum glucose ≥ 200 mg/dL or 2-h oral glucose tolerance test ≥ 140 mg/dL or HbA1c ≥ 5.7% or established diagnosis of T2DM or specific treatment for T2DM.
3. Hypertension testified by blood pressure (BP) ≥ 130/80 mmHg for age ≥ 13 years; for age < 13 years, BP ≥ 95th percentile or ≥130/80 mmHg (whichever is lower) or use of antihypertensive treatment.
4. Hypertriglyceridemia with triglyceride ≥ 100 mg/dL for age < 10 years or triglyceride ≥ 150 mg/dL for age ≥ 10 years or lipid-lowering treatment.
5. Low cholesterol HDL, i.e., HDL ≤ 40 mg/dL or lipid-lowering treatment.

The imperative to redefine the term NAFLD in the pediatric population is even more significant than in adults, as alcohol consumption is not a typical factor, considering the widespread prohibition for individuals under the age of 18 in most countries. In children with steatosis and without typical cardiometabolic risk factors, causes such as parenteral nutrition, hepatitis C, lipodystrophy, steatogenic medication such as valproate, or inborn errors of metabolism must be considered as well. Depending on the presence of cardiometabolic risk factors alone or additional factors, the possibility of MetALD or other combination etiologies is a possibility [19]. Of note, the ultimate diagnosis of MASH in children lacks reliable non-invasive biomarkers and requires liver biopsy. According to histology, a further classification includes definite and borderline zone 1 and zone 3 patterns.

## 3. MASLD Epidemiology and Natural History

Data on the prevalence of pediatric steatotic liver disease are scanty and depend largely on a difficult diagnosis. The nomenclature aspect and, as mentioned earlier, an almost complete overlap of populations exists between MASLD and NAFLD in adults [21,22,23], but this aspect is still poorly investigated at pediatric age [14]. These considerations highlight the difficulty in identifying children with hepatic steatosis, a preliminary requirement for making a diagnosis of MASLD. The prevalence of fatty liver disease in children is strongly influenced by the diagnostic methodology. According to the NAFLD terminology, indirect methods included imaging (mainly ultrasonography) or elevations in serum aminotransferase levels in at-risk subjects. For MASLD, diagnostic includes radiologic or biopsy evidence of steatosis in children with precise metabolic risk factors, although prospective studies in pediatric MASLD are lacking, so far. Results can be also influenced by the age of screening, i.e., generally after 9 years of age, although steatosis can also develop in utero [29] and cirrhosis earlier than 9 years of age [30,31]. A further aspect is the environmental context where studies are performed, i.e., different ethnic groups, community populations, or hospital setting, etc.

The estimates of NAFLD prevalence for European populations range from 1.3% to 22.5% in children aged 3–18 years, or 11% in children aged 12.4 ± 2.6 years if defined by elevated AST or ALT concentrations more than 50 IU/L [32]. In the United States, NAFLD prevalence ranges from 9.6% of all children aged between 2 and 19 years to 38.0% of obese children when fatty liver is defined by the presence of overweight (BMI ≥ 95th percentile) and elevated ALT concentrations: more than 25.8 IU/L for boys and more than 22.1 IU/L for girls [33,34]. According to the recent US National Health and Nutrition Examination Survey (2011 to 2018), elevated ALT (>22 units/L for females and >26 units/L for males) occurred in 16% of (likely NAFLD) adolescents and in 39% of obese adolescents [35]. Another large population-based study in adolescents (range 1988–1994 to 2007–2010), examined increased ALT and reported that the prevalence of suspected NAFLD has more than doubled over the past 20 years, affecting about 11% of adolescents and one-half of obese males [34]. A meta-analysis based on abnormal ALT at various thresholds and comparison with imaging reported that the estimated NAFLD prevalence was 7% and 14% in the general population (by nine studies) and in children with obesity (by 14 studies), respectively [36]. Ultrasonography has been reported to have poor sensitivity and specificity for detecting and grading hepatic steatosis in children. Although the study is not recent, based on a PubMed search of a limited number of papers, the authors found that US had positive predictive values of 47% to 62% [37]. Data on the prevalence of liver steatosis in children and adolescents examined by ultrasonography must be interpreted with caution, since further evidence is required [36]. According to the transient elastography and controlled attenuation parameter, utilizing the Health and Nutrition Examination Survey 2017–18 database, the prevalence of steatosis in US adolescents (12–18 years) was 24.2% [38]. Irrespective of the diagnostic methods employed, recent studies (which consider the differences between NAFLD and MAFLD) estimate that, in the general population, the MAFLD prevalence stood at 33.78%, while, in a specific subgroup attending child obesity clinics, it was 44.94%. When examining subgroups, the prevalence of MAFLD was notably elevated in boys as opposed to girls, registering at 36.05% versus 26.84% in the general population and 50.20% versus 35.34% in the child-obesity-clinic-based population [39].

Histological studies have been performed during autopsy studies. The prevalence of fatty liver was 9.6% overall (steatohepatitis 3%) and 38% in obese children (steatohepatitis 23%) among 742 children and adolescents in a fairly representative study of unselected population in San Diego County [33]. Race/ethnicity played a role independently of obesity, since Hispanic youths had a fivefold increase in risk for steatotic liver as compared with Black youths. White youths had intermediate risk. Another autopsy study conducted in New York City reported a lower prevalence and severity of NAFLD in black children compared with the general obese pediatric population. Hispanic children did not have a significantly increased rate of NAFLD compared with white children. This finding is most likely related to the large proportion of Caribbean Hispanic children in New York City and the lower proportion of Mexican/Central American Hispanic ethnicity compared with the San Diego County region [40]. The finding is that Caribbean Hispanic ancestry is associated with a higher percentage of African genetic admixture and appears to be protective against hepatic steatosis [41]. Studies about the natural history of children with liver steatosis diagnosed by recent MASLD criteria are still required.

## 4. MASLD Pathogenesis and Molecular Aspects

The pathogenesis and etiology of MASLD are not yet fully understood; however, they are known to be closely associated with metabolic dysfunction. The pathogenesis of MASLD appears to be multifactorial, arising from interaction among various factors such as genetic, epigenetic, environmental, and lifestyle factors [20]. MASLD is strongly associated with metabolic dysfunction, which very often develops due to unhealthy lifestyles and dietary habits. Low physical activity, a sedentary lifestyle, a Western diet, and high caloric intake contribute to the development of overweight and obesity [16], which are primary risk factors associated with MASLD [42]. Adipose tissue expansion, accompanying obesity, leads to its dysfunction. Dysfunctional adipose tissue releases molecules such as free fatty acids (FFAs), reactive oxygen species (ROS), and pro-inflammatory cytokines (leptin, IL-6, TNF-Alpha, etc.), leading to impairment in insulin-signaling pathways and subsequent insulin resistance [43]. Insulin resistance disturbs the body’s homeostatic control of several gluco-lipid parameters, because of a status of sustained hyperinsulinemia. This imbalance contributes to the development of T2DM and de novo lipogenesis, promoting dyslipidemia [44]. Moreover, the release of pro-inflammatory molecules from visceral adipose tissue contributes to the development of a state of chronic low-grade systemic inflammation, which further impairs the insulin signaling pathway [43]. Factors such as alcohol, smoking, air pollutants, gut dysbiosis, and food contaminants may contribute to MASLD development and progression [16]. This increased uptake of FFAs by the liver leads to hepatic lipid overload and their subsequent excessive accumulation within hepatocytes [45]. Hepatic intracellular lipids exert a lipotoxic effect on hepatocytes, known as “lipotoxicity”, which arises from their toxic metabolites such as lipophosphatidylcholine, ceramides, and diacylglycerol, resulting in increased oxidative stress and development of inflammation within hepatocytes, resulting in hepatocyte dysfunction and disruption of numerous physiological processes within the liver [16]. The liver plays a central role in lipid metabolism [46]. When hepatocytes become dysfunctional, they become unable to perform their normal functions, including lipid metabolism [47]. Consequently, hepatic dysfunction further leads to impaired lipid metabolism, ultimately resulting in the development of dyslipidemia, which has systemic effects [48]. Moreover, excessive lipids in the liver will further impair the insulin signaling pathway, aggravating insulin resistance and exacerbating dysregulation in lipid metabolism, notably marked by elevated triglycerides and LDL-cholesterol levels, and lowered HDL-cholesterol levels in circulation. This progression contributes significantly to the exacerbation of MASLD [49]. Hence, MASLD becomes a significant risk contributor for hepatic complications directly affecting the liver or as extra-hepatic complications [50,51].

## 5. Challenging Diagnosis of MASLD in Children and Adolescents

### 5.1. Clinical Aspects

As for adults, children with MASLD are largely asymptomatic [52], although a minority of children can describe right upper quadrant pain or vague abdominal discomfort and fatigue [53,54]. Other symptoms are even more vague and might depend on obesity-associated comorbidities, i.e., constipation, gastroesophageal reflux disease, and functional abdominal pain [55]. Other signs related to end-stage liver disease include spider angiomata, palmar erythema, muscle wasting, jaundice, or encephalopathy but they are rarely observed, as MASLD in childhood has a rare progression to decompensated cirrhosis. As a consequence of the close association with insulin resistance/T2DM, the physical exam may reveal acanthosis nigricans and abdominal adiposity. This latter finding can hide the presence of hepatomegaly and/or splenomegaly. For pediatric MASLD, epidemiological data, risk factors, natural history, and therapy are based on studies investigating children with chronically elevated liver enzymes. While fatty liver disease with normal ALT levels is acknowledged in both adults and pediatric populations [56], an increased ALT level alone may indicate liver injury but may not necessarily indicate NAFLD/MASLD. Laboratory abnormalities, if present, include elevated serum liver transaminases (alanine aminotransferase [ALT] and aspartate aminotransferase [AST]), alkaline phosphatase, and gamma-glutamyl transpeptidase (GGTP) [31,52,57,58,59].

### 5.2. Screening

In historical studies, the screening for NAFLD was based on the measurement of serum ALT [60]. Aminotransferase elevations act as a surrogate biomarker of NAFLD but still yield poor sensitivity and specificity for detecting steatosis, depending also on the ALT cut-off used in the studies [61]. For the detection of steatosis, Jebeile et al. [1] recommend evaluation of ALT concentrations (<25.8 IU/L for boys and <22.1 IU/L for girls), liver ultrasound, or transient elastography indifferently. The North American Society for Pediatric Gastroenterology, Hepatology, and Nutrition (NASPHGAN) recommends as a screening test the measurement of serum ALT as an inexpensive, minimally invasive, and universally available test [60]. However, the normal ALT cut-off varies across populations [62,63] and the sensibility of ALT measurement in overweight and obese children age ≥ 10 years, (considering ALT ≥ 50 IU/L for boys and ≥44 IU/L for girls as abnormal) is less than 90% [61].

Conventional ultrasonography, although used in clinical practice, yields poor outcomes for the diagnosis or grading of fatty liver in children [37]. The increased echogenicity can be detected by ultrasonography, but the sensitivity and specificity for detecting clinically significant liver disease are poor [60,64,65]. Ultrasonography and ALT exhibit comparable yet modest diagnostic precision (the ROC of ALT and US were not significantly different: 0.74 and 0.70, respectively) for detecting hepatic steatosis in obese children. The diagnostic accuracy of ALT (optimal threshold ≥ 40 IU/L) and ultrasonography (optimal steatosis score ≥ 2) is comparable with moderate specificity (89% for ALT, 80% for ultrasonography), and their combination did not enhance overall accuracy, suggesting limitations in current screening strategies for hepatic steatosis in children [66]. Computed tomography, while reasonably effective in sensitivity and specificity for detecting steatosis, is not advisable for diagnostic purposes due to the associated risk of radiation.

More validation studies are necessary for the assessment of liver fibrosis by transient elastography (FibroScan^®^), ultrasound shear wave elastography, or enhanced liver fibrosis testing [66]. The same applies to other imaging tests such as magnetic resonance elastography for assessment of liver stiffness and magnetic resonance imaging proton density fat fraction for estimation of liver fat content [66,67]. These techniques, due to their high cost and time-consuming nature, are primarily applicable in research settings [68]. The first guideline was issued in 2012 by the European Society for Paediatric Gastroenterology, Hepatology, and Nutrition and recommended screening with ALT and imaging due to the possibility that some children with hepatic steatosis still have normal liver enzymes [69,70].

As outlined later in 2017 by the North American Society for Pediatric Gastroenterology, Hepatology, and Nutrition NASPHGAN guidelines, the initiation of screening for hepatic steatosis is recommended with ALT alone [60]. Screening starts between the ages of 9 and 11 years for obese children (BMI ≥ 95th percentile) and overweight children (BMI ≥ 85th and <94th percentile) who exhibit additional risk factors such as central adiposity, insulin resistance, prediabetes or diabetes, dyslipidemia, sleep apnea, or a family history of NAFLD/NASH. Consideration for earlier screening is warranted for younger patients with factors like severe obesity, family history of NAFLD/NASH, or hypopituitarism. Furthermore, it is advisable to screen siblings and parents of children with NAFLD if they present known risk factors, including obesity, Hispanic ethnicity, insulin resistance, prediabetes, diabetes, and dyslipidemia [60].

The upper limit of normal (ULN) is different for adolescents of 12 to 17 years (girls—22 units/L; boys—26 units/L, referred to the 97th percentiles for a healthy lean population [71,72]) and children 1 to <12 years (30 units/L, referred to the CALIPER study [73]). After the screening, the subsequent follow-up will depend on the degree and duration of ALT elevation, ranging from 1 to 3 years to a few months, to specialist referral with normal ALT, moderate ALT elevations (ALT > ULN but <80 units/L), ALT persistently > 2 × ULN (i.e., >44 units/L for adolescent girls and 52 units/L for boys) for three or more months, respectively. Attention must be given to the onset of other viral infections, acute increase in ALT, symptoms of more advanced liver disease [74], degree of obesity, ethnicity (Hispanic children [75]), or comorbid conditions contributing to the onset or progression of MASLD, namely, obstructive sleep apnea (OSA), hypothyroidism, depression or anxiety, use of alcohol and drugs, or family history of metabolic, autoimmune liver diseases, other chronic liver diseases, insulin resistance, or T2DM (see below).

Thus, each method described above has some limitations. The ultimate diagnosis of all stages of liver steatosis relies on liver biopsy as the gold standard, but the technique is invasive, has the potential for sampling errors, and is not easily accepted by patients and their families [67]. Until new evidence accumulates about the best screening methodology in pediatric MASLD, screening with ALT in most settings will remain the main approach to select the subgroup of subjects at increased risk of progression, although the subset of children with hepatic steatosis who have normal liver enzymes will be missed. Further limitations in this context are the ULN, which can govern sensitivity and specificity [61], and the possibility of progression to fibrosis despite normal ALT (12%) or moderately elevated ALT (54%) [56].

## 6. T2DM and MASLD

### 6.1. Interconnections

**Figure 2:**
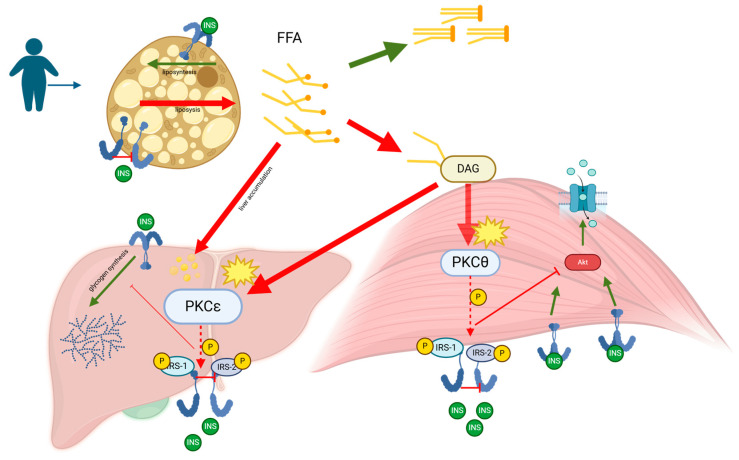
Metabolic dysfunction–associated steatotic liver disease (MASLD), formerly known as non-alcoholic fatty liver disease (NAFLD), is characterized by the accumulation of fat in the liver primarily due to metabolic dysregulation. The pathogenesis of MASLD is closely linked to insulin resistance, which plays a critical role in the progression of the disease. Elevated levels of free fatty acids (FFAs) in the bloodstream, often a result of increased lipolysis from adipose tissue in obesity and metabolic syndrome, are taken up by the liver and muscles. In the liver, these FFAs are esterified into triglycerides, leading to hepatic steatosis, the hallmark of MASLD. When the liver’s capacity to oxidize and store FFAs as triglycerides is exceeded, FFAs are diverted into alternative pathways, including the synthesis of diacylglycerol (DAG). Within hepatocytes, FFAs are esterified with glycerol-3-phosphate to form DAG, which acts as a second messenger to activate specific isoforms of protein kinase C (PKC), such as PKCε in the liver. The activation of PKCε results in its translocation to the cell membrane, where it phosphorylates serine/threonine residues on insulin receptor substrates (IRS-1 and IRS-2), impairing insulin signaling pathways. This impairment leads to reduced activation of Akt, decreased glycogen synthesis, and increased gluconeogenesis, contributing to hyperglycemia and worsening insulin resistance. As MASLD progresses, the chronic overload of FFAs enhances triglyceride synthesis and fatty acid oxidation, resulting in the production of reactive oxygen species (ROS). These ROS cause oxidative stress and cellular damage, triggering inflammatory responses that can escalate the disease from simple steatosis to non-alcoholic steatohepatitis (NASH). The chronic inflammation associated with NASH stimulates the production of pro-inflammatory cytokines and chemokines, which attract inflammatory cells to the liver, further aggravating hepatic injury. Over time, the persistent inflammation and oxidative stress can lead to the development of fibrosis as the liver attempts to repair the ongoing damage. This fibrotic process can progress to cirrhosis, where the liver structure is severely compromised, leading to a decline in liver function and an increased risk of hepatocellular carcinoma. Thus, MASLD represents a spectrum of liver disease that ranges from simple hepatic steatosis to varying degrees of fibrosis, cirrhosis, and potential liver failure.

Diabetic children and adolescents are a particularly monitored population, given the high association between NAFLD/MAFLD and T2DM [76,77]. Indeed, MASLD is particularly common in children living with T2DM and/or obesity. The progression of NAFLD to NASH is influenced by insulin resistance and T2DM (Figure 2). In adults, Younossi et al. [76] demonstrated an increased prevalence of cirrhosis in 132 adult subjects with histologically confirmed NAFLD and T2DM compared to those without diabetes (25% vs. 10%, respectively). The development of hepatocellular carcinoma in adults with NAFLD is influenced by T2DM acting as an independent risk factor [78]. Whether such an effect will become apparent in young populations (pediatric patients, adolescents) deserves much attention. A mild increase in serum ALT is common in T2DM adolescents [79]. In addition, obese children often suffer from insulin resistance and T2DM [80] and develop an increased accumulation of intrahepatic fat with progression to liver steatosis [77]. According to the TODAY study, 6.5% of youths exhibited a mild ALT elevation (1.5 to 2.5 times the upper limits of normal) shortly after being diagnosed with T2DM. Over the course of the study (2 to 6.5 years of follow-up), 16% of participants experienced ALT elevations [81].

As for screening, the International Society for Pediatric and Adolescent Diabetes (ISPAD) has developed specific recommendations for prompt NAFLD detection in children with T2DM [82]. In T2DM children and adolescents, liver enzymes ALT and AST should be measured at diagnosis and annually thereafter, and sooner if abnormal. In addition, if liver enzymes remain > 3 times the upper limit of normal after 6 months, patients must be seen by a pediatric gastroenterologist for consultation to exclude other causes of elevated liver enzymes and to consider imaging and/or liver biopsy. Vice versa, NAFLD is often associated with metabolic disturbances.

In general, children with biopsy-proven NAFLD suffer from a higher prevalence of impaired fasting glucose, impaired glucose tolerance, and T2DM compared to children without NAFLD of similar age, gender, and adiposity [83]. The prevalence of prediabetes or diabetes in children with biopsy-proven NAFLD is 20–30% in Italy and in the United States [83,84]. Normoglycemic children with biopsy- or imaging-proven NAFLD in Israel had a 3-fold increased risk for T2DM compared to youths without NAFLD. This difference was evident after adjustment for BMI [85]. In a meta-analysis, adults with T2DM had an increased prevalence of NAFLD (55.5%), NASH (37%), and advanced fibrosis (17%) [86]. The prevalence of T2DM and prediabetes in children with NAFLD is 23.4% and 6.5%, respectively. The prevalence of NASH is higher in children with T2DM (43.2%) compared to children with prediabetes (34.2%) or normal glucose tolerance (22%). Girls with NAFLD have a higher risk of having prediabetes and T2DM than boys with NAFLD [84]. Children with NAFLD exhibit notably higher rates of impaired fasting glucose when compared to matched controls who are overweight or obese [87]. Increased intrahepatic fat content is linked to more pronounced insulin resistance and impaired glucose regulation before the development of clinically overt diabetes [87,88]. D’Adamo et al. [87] demonstrated a central role of hepatic fat content in the pathogenesis of insulin resistance in obese adolescents independently of visceral fat and intrabdominal intramyocellular lipid content. However, whether hepatic steatosis is a consequence or a cause of the metabolic derangements in insulin sensitivity is still debatable [61]. The combined impact of MASLD and T2DM heightens the risks of morbidity and mortality in childhood [89]. In particular, T2DM in MASLD becomes a strong independent predictor of progression to inflammation and fibrosis, namely MASH [90].

### 6.2. Follow-Up Implications

The progression of pediatric fatty liver disease is not fully elucidated. Similarly to adults, children with NAFLD and T2DM likely have an increased risk of progression to NASH and cirrhosis [84]. Data on the natural history of pediatric NAFLD emerge from a study of 122 children with biopsy-confirmed NAFLD, who enrolled in the placebo arm of clinical trials and received standard-of-care lifestyle counseling. One-third of participants experienced histological progression after a median of 1.6 years of follow-up, and this progression correlated with the severity of insulin resistance [91]. Repeat liver biopsy revealed that borderline/definite NASH at baseline ameliorated to no NASH (29%), fatty liver or borderline NASH at baseline showed progression to definite NASH (18%), NAFLD resolved in 2.4% percent (all children with steatosis but not NASH at baseline), and fibrosis improved (34%) and progressed (23%). Clinical characteristics associated with disease progression/fibrosis worsening included baseline adolescent age, ALT, and total and low-density lipoprotein (LDL) cholesterol levels. Longitudinal predictors of disease progression were a rising ALT, gamma-glutamyl transpeptidase (GGTP), and hemoglobin A1c, as well as the development of type 2 diabetes. These factors may help guide escalation to more intensive interventions and decisions regarding repeat liver biopsy. Overall, 7% of the cohort developed incident T2DM within two years, at a cumulative incidence rate nearly 300-fold the rate of the general pediatric population. Similarly, children with congenital generalized lipodystrophy, characterized by very high insulin resistance, exhibit advanced fatty liver disease and the complete spectrum of features associated with metabolic syndrome [92]. The strong association between T2DM and MAFLD, along with the rapid progression of the latter towards advanced liver diseases, especially in diabetic patients, underscores the imperative for pediatric endocrinologists to actively investigate the presence of MASLD in children with T2DM. We contend that ISPAD recommendations, advocating for steatosis screening through liver enzymes at the onset of the pathology and subsequently on an annual basis, may prove insufficient. The challenging determination of ALT normality cut-offs and their relative sensitivity and specificity could compromise the identification of children with MASLD in a significantly high-risk population. Likely, children who do not achieve adequate glycemic control and satisfactory weight loss in the year following the diagnosis of T2DM, and with normal liver enzyme testing, are candidates for hepatic MRI assessment.

### 6.3. Therapeutic Aspects

Most data at pediatric age derive from studies looking at therapeutic outcome in NAFLD or NASH (Table 1). Weight management by diet and exercise remains the only established treatment for NAFLD [30,60]. In selected adolescents with severe obesity, bariatric surgery may be appropriate [30]. A large meta-analysis based on 106 randomized controlled trials, with four conducted in children, found limited evidence for therapy in NAFLD, with no specific recommendations [93]. A systematic review in 2617 NAFLD individuals with and without T2DM reported that few antidiabetic medications improve liver enzymes, but only glitazones and glucagon-like peptide 1 receptor (GLP-1) agonists, liraglutide and semaglutide, appear to improve NAFLD histology. Longer trials are required in this field [94]. While the presence of NAFLD does not preclude the use of metformin, achieving optimal blood glucose levels and improving weight do contribute to amelioration of NAFLD. Concerning specific NAFLD pharmacotherapy, to date, no medications are recommended for routine treatment of MASLD in children [60], including vitamin E, metformin, losartan, and cysteamine bitartrate. None of such treatments is superior to lifestyle intervention [95,96]. Such evidence underscores the key role of healthy lifestyle interventions in promoting weight loss and optimizing blood glucose levels to improve NAFLD [97].

In addition to lifestyle interventions, dietary regulation plays a crucial role in the management of MASLD in children (Table 2). Overnutrition, particularly excessive intake of fats and sugars, is closely linked to the pathogenesis of obesity, diabetes, and MASLD [110]. Nutritional interventions should not only focus on reducing calorie intake but also on modifying the overall nutrient composition. Diets low in saturated fats, fructose, or sucrose-containing beverages and high in unsaturated fats, as well as those rich in dietary fiber, have been shown to improve metabolic function, reduce liver fat accumulation, and decrease inflammation [111]. Specific dietary patterns, such as the Mediterranean diet, which is rich in fruits, vegetables, whole grains, and healthy fats, have been associated with reduced hepatic steatosis and improved insulin sensitivity in pediatric populations [112]. Moreover, the potential impact of high-fat, low-fiber diets on gut microbiota composition, particularly the increased Firmicutes/Bacteroidetes ratio, has been highlighted by various association studies. The mechanisms by which these microbes may contribute to MASLD progression include damage to the gut vascular barrier, a shift toward a less tolerant immune environment, and harmful metabolic alterations. These alterations include a relative decrease in propionate and butyrate in favor of acetate, endogenous ethanol production, and disruption of the FXR-mediated gut-liver axis driven by unconjugated bile acids. These findings emphasize the importance of dietary interventions not only in improving metabolic function but also in modulating gut microbiota composition, which may ultimately influence the development and progression of MASLD [113].

## 7. T1DM and MASLD

Another significant consideration in the management of hepatic steatosis is the involvement of Type 1 diabetes mellitus (T1DM). While the majority of research focuses on T2DM in relation to MASLD, recent studies have highlighted that patients with T1DM can also develop hepatic steatosis and potentially progress to NASH [122].

The pathophysiological mechanisms linking T1DM to hepatic steatosis include insulin resistance, which, although less prevalent in T1DM compared to T2DM, can still occur, especially in patients with long-standing diabetes or those who are overweight [122]. Additionally, chronic inflammation associated with T1DM may contribute to liver damage and steatosis [123].

For screening MASLD in patients with T1DM, it is essential to consider several factors. Current guidelines suggest that patients with T1DM should be screened for hepatic steatosis if they have a history of obesity or dyslipidemia, or if their glycemic control is suboptimal [124]. Non-invasive methods such as serum biomarkers (e.g., FIB-4, NAFLD Fibrosis Score [NFS], and APRI) and imaging techniques (e.g., ultrasonography or elastography) are recommended for initial evaluation. Regular monitoring and early intervention are crucial, particularly for patients exhibiting signs of liver fibrosis or progressive liver disease.

Careful and proactive management of hepatic health is necessary for individuals with T1DM to prevent potential progression to more severe liver conditions. Coordinated care involving endocrinologists and hepatologists can help in tailoring individualized screening and management plans for these patients [124].

## 8. Conclusions

The continuous rise in childhood obesity and T2DM has increased the attention of researchers towards potential liver implications. The concern is mainly based on the rationale that metabolic-dysfunction-associated conditions become chronic, long-lasting, and potentially harmful events in the lives of young populations. The close interactions between cardiometabolic risk abnormalities and MASLD will expose young individuals to a double risk, i.e., early cardiovascular- and late liver-related.

This is particularly true in both Westernized societies and emerging societies in developing countries where the effects of bad lifestyles, including obesogenic diets and sedentary habits, will affect the healthy status of several societies.

In this respect, pediatric populations and their families will require close surveillance, now and in the future, to prevent or to actively treat T2DM, overweight, obesity, metabolic syndrome, and steatotic liver. Current screening methods for steatotic liver, such as ALT measurement and ultrasonography, exhibit limitations, emphasizing the need for precise diagnostic strategies, including hepatic MRI, to improve outcomes in this high-risk pediatric population.

Taken together, the battle against obesity, especially in early ages, is becoming more and more challenging. Thus, high medical, social, and economic consequences and potentially serious long-term sequelae are anticipated. This truth also stands for the rising prevalence of steatotic liver disease in children with metabolic disorders. We need more confidence and new actions to reverse such unfavorable trends.

## Figures and Tables

**Table 1:** Phase II, III, and IV clinical trials for MASLD treatment in children.

Phases	Clinical Trials.gov	Start Date of Trial (Day Month Year)	Drug	Molecular Mechanism (Target)	Patients	Main Findings	Adverse Effect	References
II	NCT02201160	1 January 2009	n-3 PUFA	anti-inflammatory, insulin metabolism regulator	NAFLD young male	FLI, ALT, and ALT/AST ratio reduction, lipid profile, and carotid intima-media thickness improvement	No	[98]
I–II	NCT00885313	1 March 2009	Docosahexanoic Acid	anti-inflammatory	NAFLD children	improvement in liver steatosis by US	No	[99]
II–III	NCT01529268	1 June 2012	cysteamine bitartrate	activators of PPARα	children with NAFLD activity scores ≥ 4	AST, ALT, and lobular inflammation reductions	No	[100]
III	NCT02098317	1 January 2014	Docosahexanoic Acid + Vitamin D	anti-inflammatory, immunity regulation	children and adolescents biopsy-proven NAFLD	improvement in IR, lipid profile, ALT, and NAFLD activity score	No	[101]
II	ChiCTRIPR-17011267	1 March 2017	rhGH	stimulation of growth, IGF-1 production	NAFLD and obese boys	reduction in liver enzymes, CRP, BMI, LDL-C. Increase in HDL-C	No	[102]
III	NCT02842567	1 April 2017	hydroxytyrosol + VitE	antioxidant, anti-inflammatory	children and adolescents biopsy-proven NASH	increase IL-10	No	[103]
III	PACTR201710002634203	19 October 2017	Vit D	anti-inflammatory and insulin-sensitizing effects	children with biopsy-proven NAFLD	improvement in hepatic steatosis, liver enzymes, cholesterol	No	[104]
III	NCT03467217	2 October 2018	Losartan	angiotensin II receptor blocker	histologic NAFLD adolescents NAFLD activity score ≥ 3, and (ALT) ≥ 50 U/L.	reduction of ALT	No	[105]
II	IRCT20170628034786N2	16 January 2019	l-carnitine	Transport of fatty acids into mitochondria	NAFLD children	no impact on liver enzymes	No	[106]
II	NCT04165343	1 February 2020	Elafibranor	Dual PPARα/δ agonist	NASH Children	ALT reduction	No	[107]
III	IRCT20200531047614N1	1 September 2020	elemental zinc	anti-inflammatory and antioxidant	NASH overweight or obese children and adolescents	ALT, CRP reduction, HDL-cholesterol increase	No	[108]
II	IRCT20220409054467N2	13 May 2022	Orlistat	inhibiting pancreatic lipase, reducing the absorption of dietary fats	NAFLD and overweight/obese adolescents	improvement in liver enzymes, steatosis, glucose/lipid metabolism	greasy stools, sleep problems, weakness, headache	[109]

Legend: ALT, alanine aminotransferase; AST, aspartate aminotransferase; BMI, body-mass index; CRP, C-reactive protein; DHA, docosahexaenoic acid; FLI, fatty liver index; HDL, high-density lipoprotein; IGF-1, insulin-like growth factor 1; IL-10, interleukin 10; IR, insulin resistance; LDL, low-density lipoprotein; NASH, non-alcoholic steatohepatitis; NAFLD, non-alcoholic fatty liver disease; PUFA, polyunsaturated fatty acids; PPAR, peroxisome proliferator-activated receptor; rhGH, recombinant human growth hormone; US, ultrasonography.

**Table 2:** Lifestyle and dietary intervention for MASLD in children.

Intervention	Subjects	Main Findings	References
1 year of normocaloric balanced diet and physical exercise	26 obese children	Reduction from 36.4% to 7.7% of steatosis.	[114]
8 weeks of diet low in free sugar vs. usual diet	40 obese adolescents	Greater reduction in hepatic steatosis in the low free sugar diet group.	[115]
12 weeks of MD vs. LFD	44 obese adolescents	Improvement of steatosis and IR in both groups. Reduction of AST, CRP, and IL-6 in the MD group.	[116]
22-week lifestyle intervention with or without intensive exercise	102 overweight/obese children	Reduction in steatosis, weight, BMI, and GGT was higher in the intensive exercise group.	[117]
6 months of low-glycemic-load vs. conventional LFD and nutrition education and behavioral counseling of equal intensity	16 obese children	Reduction of steatosis in both groups.	[118]
8 weeks of dietary sugar restriction vs. usual diet	29 adolescents with MASLD	Hepatic de novo lipogenesis, hepatic fat content and fasting insulin was significantly decreased in the treatment group.	[119]
4 months of High-protein (PRO) vs. High-carbohydrate (CHO) diet	13 adolescents	PRO group increased short-chain acylcarnitine levels, reduced liver fat, and preserved lean mass, while the CHO group lost lean mass. Both groups maintained metabolic control.	[120]
Moderately CHO-restricted diet (CRD) vs. fat-restricted diet (FRD) for 8 weeks	32 children/adolescents	The CRD group showed a significant reduction in hepatic lipid (−6.0 ± 4.7%), and greater improvements in insulin resistance, abdominal fat mass, and body fat mass compared to the FRD group.	[121]

Legend: AST, aspartate aminotransferase; BMI, body-mass index; CHO, carbohydrate; CRD, carbohydrate-restricted diet; CRP, C-reactive protein; FRD, fat-restricted diet; FXR, farnesoid X receptor; GGT, gamma-glutamyl transferase; IL-6, interleukin 6; IR, insulin resistance; LFD, low-fat diet; MD, Mediterranean diet; MASLD, metabolic dysfunction–associated steatotic liver disease; PRO, protein.

## Data Availability

Not applicable.
